# Prevalence and Trajectories of Perinatal Anxiety and Depression in a Large Urban Medical Center

**DOI:** 10.1001/jamanetworkopen.2025.33111

**Published:** 2025-09-22

**Authors:** Nili Solomonov, Daniel Kerchner, Yinglin Dai, Minhee Kwon, Delaney G. Callaghan, Maddy M. Schier, Yiye Zhang, Lauren M. Osborne, Natalie C. Benda

**Affiliations:** 1 Department of Psychiatry, Weill Cornell Medicine, New York, New York; 2 George Washington University Libraries, George Washington University, Washington, DC; 3 Department of Biostatistics and Bioinformatics, George Washington University, Washington, DC; 4 Department of Population Health Sciences, Weill Cornell Medicine, New York, New York; 5 Division of Health Information, Department of Population Health Sciences, Weill Cornell Medicine, New York, New York; 6 Department of Emergency Medicine, Weill Cornell Medicine, New York, New York; 7 Department of Obstetrics and Gynecology, Department of Psychiatry, Weill Cornell Medicine, New York, New York; 8 Columbia University School of Nursing, New York, New York

## Abstract

**Importance:**

Perinatal depression and anxiety are common yet underdiagnosed and undertreated. There are limited data on screening rates, severity, and treatment, and thus an urgent need to estimate accurately perinatal depression and anxiety over time, to inform timely and efficacious interventions.

**Objective:**

To evaluate screening and treatment rates, prevalence, and symptom trajectories over time of perinatal depression and anxiety in a large urban medical center.

**Design, Setting, and Participants:**

This retrospective cohort study included electronic health records of all patients who gave birth at NewYork-Presbyterian (NYP)/Weill Cornell Medical Center or NYP Lower Manhattan Hospital between December 1, 2020, and February 1, 2024. In March 2023, a mandatory Edinburgh Postnatal Depression Scale (EPDS) screening policy was implemented in 3 clinics covering 35% of deliveries in the hospital.

**Exposure:**

Completion of mental health screening during the perinatal period.

**Main Outcomes and Measures:**

The study included 3 primary mental health measures: depression severity (Patient Health Questionnaire–9 [PHQ-9]), anxiety severity (Generalized Anxiety Disorder–7 [GAD-7]), and perinatal depression severity (EPDS). Percentage of women screened, prevalence of clinically meaningful symptoms, as well as rates and frequency of mental health services were examined. Furthermore, symptom changes 1 year before and after delivery were tested using mixed effects models, and whether patient characteristics and mental health services were associated with symptom change was determined.

**Results:**

The study included data from medical records of 27 393 women who gave birth during the study period. The final sample included 3051 women (mean [SD] age, 34.3 [5.2] years; age range, 14-54 years) who completed perinatal depression or anxiety screening within 1 year before and after birth. A total of 723 women (3.0%) were administered depression screening (PHQ-9), and 472 (2.0%) completed anxiety (GAD-7) screening. Following a mandatory screening policy, EPDS screening rates increased from 1.0% (274 women) before March 2023 to 14.2% (2304 women) after March 2023. Of those screened, 23.2% (95% CI, 21.7%-24.8%) reported clinically meaningful depression symptoms and 8.8% (95% CI, 7.2%-10.8%) endorsed suicidality; 17.1% of women (523 women) screened received mental health services. Treated women were mostly seen 4 months before and after birth for psychosocial interventions, had faster depression reductions over time (PHQ-9, *F*_1,1504_ = 9.6; *P* = .002), and a sustained decline in depression severity postpartum, compared with untreated women (*F*_1,5166_ = 33.8; *P* < .001).

**Conclusions and Relevance:**

These findings underscore the need for routine and consistent screening, monitoring, and treatment of perinatal depression and anxiety. Women who received mental health services had faster reductions in depression over time, highlighting the potential impact for scalable and efficacious interventions during this critical period.

## Key Points

**Question:**

What are the rates of screening, treatment, and prevalence of clinically meaningful perinatal depression and anxiety over time in a large urban medical center?

**Findings:**

Among 27 393 perinatal women, only 3.0% to 14.2% were screened for anxiety and depression, and in clinics with mandatory screening, 23.2% of women reported clinical depression symptoms and 8.8% reported suicidality. Women who received mental health services (17.1%) showed a more rapid depression reduction and sustained gains, compared with untreated women.

**Meaning:**

These findings suggest that there is a need for routine mental health screening, monitoring, and timely interventions to improve risk detection and reduce perinatal depression and anxiety.

## Teaser

This cohort study examines the screening and treatment rates, prevalence, and symptom trajectories of perinatal depression and anxiety among patients at a large urban medical center.

## Introduction

Perinatal depression and anxiety affect approximately 12%^1^ and 20%^2^ of women, respectively, and remain underdiagnosed and undertreated, with only a fraction of women receiving treatment.^3^ These disorders are associated with increased risk for adverse birth outcomes, poor maternal health, pregnancy-related death, social relationship problems, and disruptions in infant and child development.^4^ There is an urgent need to accurately estimate the screening rates, prevalence, and persistence of perinatal depression and anxiety in clinical practice settings, to inform timely and efficacious interventions.

Routine screening for perinatal depression and anxiety can reduce risk of persistence and poor mental health outcomes for mothers and infants and is recommended by the American College of Obstetricians and Gynecologists across the perinatal period.^5^ However, most perinatal women are not screened for mental health disorders, with 50% to 70% of symptomatic women remaining without a diagnosis.^6,7,8^ Reasons for lack of screening include clinician time constraints, resource availability, insufficient training for obstetrics practitioners,^9^ negative attitudes toward mental health and screening tools, and women’s fear of stigma and reluctance to report symptoms.^10^ Even when women are screened, most are not monitored routinely through 12 months after birth^11^ and are lost to follow-up in the system.^12^

Estimates of depression and anxiety prevalence vary dramatically across studies, but consistently indicate that when women are screened, 15% to 20% endorse clinically meaningful symptoms of perinatal depression^1,12,13^ and anxiety.^12,13,14^ Mandatory routine screening policies to track anxiety and depression during the perinatal period can improve risk detection and timely interventions.^15^ Yet these policies are rarely implemented broadly in large hospital systems.^8,11^

Psychosocial interventions are efficacious for preventing and treating perinatal depression and anxiety^16,17^ and are preferred over medication by two-thirds of perinatal women.^18,19,20^ More than one-half of those reporting symptoms experience a first-time depressive episode without comorbid substance use or personality disorders,^21^ and are, thus, ideal candidates for first-line psychosocial interventions. However, only a fraction of women receive treatment.^3^ Barriers include socioeconomic challenges, poor access, limited coordination between obstetrics and psychiatry, stigma, fear of custody loss, and concerns about medication effects on the fetus and infant.^22,23,24,25^

Prior studies estimating the prevalence of screening and treatment of perinatal depression and anxiety have mostly focused on small samples,^26,27,28^ with few large-scale studies in recent years.^29^ Electronic health records (EHRs) offer a unique opportunity to estimate prevalence and severity of symptoms in large representative samples,^30^ improve risk detection, and inform intervention development for this vulnerable time period.^31,32^ Here, we present data from one of the largest hospital systems in the country in a diverse urban area.

This study aimed to estimate routine screening rates, severity, and trajectories of perinatal depression and anxiety in a diverse sample in a large urban hospital system. The sample included women who gave birth in the system. Our aim was 2-fold: first, to evaluate screening rates and prevalence of clinically significant perinatal depression and anxiety, and how these changed following a transition from practitioner-initiated to mandatory screening; and second, to examine symptom trajectories over time and test the associations of risk factors and mental health services with changes in symptoms.

## Methods

### Study Design and Setting

This is a retrospective cohort study of perinatal depression and anxiety for women who gave birth in the NewYork Presbyterian Hospital system between December 1, 2020, and February 1, 2024. The Weill Cornell Medicine’s institutional review board approved the study. In compliance with Weill Cornell institutional review board policy, a waiver of informed consent was obtained for this retrospective study because it posed no more than minimal risk; did not affect patient care, rights or welfare; involved data collected after discharge without any patient contact; and was deidentified for the purpose of analyses. This study followed the Strengthening the Reporting of Observational Studies in Epidemiology (STROBE) reporting guidelines for cohort studies.^33^

All data were acquired from Epic Systems, implemented at the hospital in October 2020 across the inpatient and outpatient clinical services. Self-reported data included the Patient Health Questionnaire–9 (PHQ-9),^34^ the Edinburgh Postnatal Depression Scale (EPDS),^35^ and the Generalized Anxiety Disorder Questionnaire–7 (GAD-7).^36^

Before March 1, 2023, screening was guided by clinical judgment, with measures administered most often when women presented with mental health concerns. Documentation of measures included paper-based forms completed in the clinic and patient self-reports completed online via the patient portal or during in-person or remote visits.

Beginning March 1, 2023, as part of a pilot program, mandatory EPDS screening for all women was implemented in 3 of 8 obstetrics and gynecology clinics, covering 35% of deliveries in the hospital system. Screening was required at the initial prenatal visit, the 28th week visit, and the 6-week postpartum visit, and was automated through Epic. Women could not opt out of screening and had to complete the EPDS before or during their visit. Thus, the pilot eliminated selection bias in screenings from both the practitioner and the patient. Implementation was gradual, because of early challenges with the automation of measures administration, which were resolved within 6 months.

### Participants

We analyzed data from medical records of women who gave birth during the study period. Our final sample included women who completed a depression or anxiety screening within 1 year before or after delivery (eTable 1 in Supplement 1). We excluded data outside this period to focus on the key perinatal time frame.

### Data Sources and Measures

Data were extracted from Epic EHR metadata collected during routine clinical care. We analyzed patients’ demographic and clinical characteristics, mental health screening measures, and mental health visits. Race and ethnicity were self-reported, recorded in medical records, and included American Indian or Alaska Native, Asian, Black or African American, Hispanic, White, and other, which includes Ashkenazi Jewish, Native Hawaiian or Other Pacific Islander, and other combinations not described. Data on race and ethnicity are included to examine their role in screening, prevalence, and treatment of perinatal depression and anxiety. Mental health visits included those from psychology, psychiatry, social work, and obstetric and gynecology services, where embedded mental health practitioners deliver care. Visits were filtered by primary billable diagnosis for a mental health condition. The study included 3 primary mental health measures: depression severity (PHQ-9),^34^ perinatal depression severity (EPDS),^35^ and anxiety severity (GAD-7).^36^

### Statistical Analysis

Baseline demographics and clinical characteristics were collected at delivery. Maternal age was calculated from date of birth. We summarized data using means and SDs for continuous variables, and frequencies and percentages for categorical variables. When mental health measures were present at more than 1 time point, we created a time series for each woman from data spanning 1 year before and after birth. We defined the day of birth as time 0 and included all data available from 1 year before (ie, day −365; prenatal period) to 1 year following delivery (ie, day 365; postpartum period). We compared the included screened women and excluded unscreened women (eTable 2 in Supplement 1).

#### Screening Rates and Severity of Depression and Anxiety

For screening rates, we combined completed screenings for each measure (PHQ, GAD-7, and EPDS). We calculated the mean number of assessments during the perinatal and postpartum periods, the mean date completed and spacing between assessments.

We assessed severity and prevalence of clinically meaningful symptoms, by computing the peak severity for each woman during the study period. Furthermore, we calculated the yearly frequency and percentage of women who endorsed clinically meaningful symptoms (a score of ≥10 on any of the 3 measures at any point during the study period).

We assessed the influence of selection bias in screening on our prevalence results by comparing women screened before and after the mandatory EPDS implementation (March 1, 2023), examining demographics and clinical characteristics (eTable 1 in Supplement 1), prevalence of clinically meaningful symptoms (eFigure 1 in Supplement 1), and changes in symptoms over time (eFigure 2 in Supplement 1).

#### Mental Health Visits

We calculated the percentage of women receiving mental health services and the number of visits delivered. We also examined patterns of mental health services delivery by calculating the mean number, timing, and spacing of visits during the prenatal and postpartum periods, in the overall sample and within women screened with each measure (PHQ-9, EPDS, and GAD-7).

#### Trajectories of Severity Over Time

For each woman, we constructed a time series to estimate change over time on each of the measures (PHQ-9, EPDS, and GAD-7). We did not apply imputations to reduce risk of estimation bias. We excluded observations with missing items and, thus, without a total score on each scale. We then entered the time series data into mixed-effects models to test changes over time. For visualization, we divided the sample into women with (a score of ≥10 on a scale) and without (score <10 on a scale) serious symptoms.

Using mixed-effects models, we examined symptom change 1 year before and after delivery. We tested whether patient characteristics and mental health visits were associated with symptoms changes over time. Because of an imbalance in number of mental health visits, we included visits as a binary variable (yes or no). Models included participant-level random intercept, fixed effects for time and patient characteristics, and a time-by-mental health visits interaction. Independent variables with greater than 95% complete data included age, ethnicity, race, marital status, months from birth, and mental health visits. We compared Akaike information criterion and Bayes information criterion for linear vs quadratic models with 1-way analysis of variance and selected the best fitted model. For GAD-7 and EPDS, the quadratic models fitted best. For PHQ-9, the linear and quadratic models did not statistically significantly differ. Thus, we report the more parsimonious linear model. Data were analyzed using R statistical software version 4.4.3 (R Project for Statistical Computing). All *P* values were 2-sided with a significance threshold of *P* < .05.

## Results

**Figure 1.**
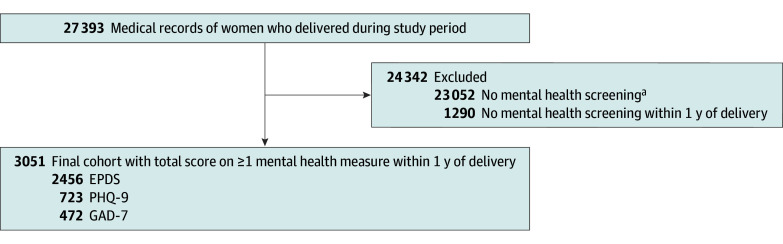
Patient Enrollment Flowchart EPDS indicates Edinburgh Postnatal Depression Scale; GAD-7, Generalized Anxiety Disorder–7 scale; and PHQ-9, Patient Health Questionnaire–9 scale. ^a^An incomplete screening or a screening with no total score is categorized as no screening.

The overall sample included 27 393 women who gave birth in our hospital system. We excluded 22 544 with no mental health screening, 508 with incomplete data, and 1290 with screening outside of the perinatal window (Figure 1). Our analyses focused on a sample of 3051 women (mean [SD] age, 34.3 [5.2] years; age range, 14-54 years) who completed mental health assessments within 1 year before or after delivery, with 850 assessments before March 2023 and 2415 assessments after March 2023; 214 women completed assessments both before and after March 2023. Twelve women (0.4%) were American Indian or Alaska Native, 497 (17.0%) were Asian, 321 (11.0%) were Black or African American, 482 (16.0%) were Hispanic or Latina, 1506 (51.0%) were White, 399 (13.0%) were other race, and 222 (7.5%) declined to answer. Most individuals were married (2329 patients [79.0%]), 2528 (83.0%) had no mental health visits, 403 (13.2%) had 1 to 10 mental health visits, 105 (3.4%) had 11 to 30 visits, and 15 (0.5%) had more than 30 visits. Most women delivered vaginally (1845 patients [60.6%]) or by cesarean delivery (1145 patients [38.0%]) (Table 1).

**Table 1.** Demographics, Mental Health Visits, and Delivery Type for the Sample at Delivery (Baseline)

Characteristics	Patients, No. (%)			
	Overall (N = 3051)	PHQ-9 (n = 723)	EPDS (n = 2456)	GAD-7 (n = 472)
Race				
American Indian or Alaska Native	12 (0.4)	5 (0.7)	8 (0.3)	2 (0.4)
Asian	497 (17.0)	96 (14.0)	428 (18.0)	80 (17.0)
Black or African American	321 (11.0)	104 (15.0)	234 (9.8)	36 (7.8)
White	1506 (51.0)	286 (41.0)	1268 (53.0)	271 (58.0)
Other^a^	399 (13.0)	142 (21.0)	283 (12.0)	42 (9.1)
Declined	222 (7.5)	58 (8.4)	171 (7.1)	33 (7.1)
Unknown^b^	94	32	64	8
Ethnicity				
Hispanic or Latino	482 (16.0)	181 (26.0)	333 (14.0)	56 (12.0)
Not Hispanic or Latino	2475 (84.0)	510 (74.0)	2059 (86.0)	408 (88.0)
Unknown^b^	94	32	64	8
Marital status				
Married	2329 (79.0)	474 (69.0)	1954 (82.0)	392 (85.0)
Single	336 (11.0)	126 (18.0)	222 (9.3)	30 (6.5)
Domestic partner or significant other	268 (9.1)	84 (12.0)	198 (8.3)	38 (8.2)
Divorced or separated	9 (0.3)	4 (0.6)	6 (0.3)	2 (0.4)
Other	4 (0.1)	1 (0.1)	3 (0.1)	0
Unknown^b^	105	34	73	10
Age, mean (SD), y^c^	34.3 (5.2)	33.4 (5.4)	34.6 (5.1)	35.5 (4.4)
Unknown^b^	94	32	64	8
No. of mental health visits				
0	2528 (83.0)	469 (65.0)	2114 (86.0)	95 (20.0)
1	179 (5.9)	40 (5.5)	146 (5.9)	95 (20.0)
2-10	224 (7.3)	119 (16.0)	134 (5.5)	179 (38.0)
11-30	105 (3.4)	85 (12.0)	52 (2.1)	92 (19.0)
>30	15 (0.5)	10 (1.4)	10 (0.4)	11 (2.3)
Delivery type^d^				
Cesarean delivery	1145 (38.0)	250 (35.0)	941 (38.0)	183 (39.0)
Spontaneous abortion or therapeutic abortion	4 (0.1)	1 (0.1)	3 (0.1)	0
Vacuum	16 (0.5)	1 (0.1)	15 (0.6)	2 (0.4)
Vaginal, instrument-assisted	48 (1.6)	9 (1.2)	40 (1.6)	7 (1.5)
Vaginal, spontaneous	1797 (59.0)	449 (62.0)	1427 (58.0)	275 (58.0)
Vaginal birth after cesarean delivery	40 (1.3)	13 (1.8)	30 (1.2)	4 (0.8)
Unknown^b^	1	0	0	1

Abbreviations: EPDS, Edinburgh Postnatal Depression scale; GAD-7, Generalized Anxiety Disorder–7 scale; PHQ-9, Patient Health Questionnaire–9 scale.

^a^ Includes Ashkenazi Jewish, Native Hawaiian or Other Pacific Islander, and other combinations not described.

^b^ Unknown values were not included in calculations of percentages.

^c^ Refers to age at first birth in the study period.

^d^ For multiple birth deliveries (n = 7) with differing delivery types, 1 delivery type was arbitrarily chosen for reporting.

We compared 24 342 unscreened and 3051 screened women. The screened sample was more diverse, with more Hispanic or Latina women, fewer married women, and higher rates of cesarean deliveries and mental health services (eTable 1 in Supplement 1). We also compared women who completed the EPDS before and after mandatory EPDS implementation (March 1, 2023). After implementation, the sample was more diverse (fewer White and Asian women and more Hispanic or Latina women), was younger, and had lower rates of cesarean deliveries and mental health visits (eTable 2 in Supplement 1).

### Screening Rates and Severity of Depression and Anxiety

We found that 723 women (3.0%) completed the PHQ-9, 2456 (9.0%) completed the EPDS (274 [1.0%] before March 2023 and 2304 [14.0%] after March 2023), and 472 women (2.0%) completed the GAD-7. All analyses were then conducted in the sample of women who received mental health screening. Women who were screened completed a mean (SD) of 2.3 (2.7) prenatal PHQ-9 assessments and 3.4 (4.2) postpartum assessments, with approximately 1 month between assessments. The PHQ-9 was administered, on average, at 4.7 months before and 5.4 months after birth. Similarly, women completed a mean (SD) of 2.1 (1.5) prenatal and 1.7 (4.2) postpartum EPDS assessments, on average 3 months before and 1 month after birth, with approximately 2 months between assessments. For the GAD-7, women completed a mean (SD) of 4.4 (4.1) prenatal and 4.9 (5.0) postpartum assessments, on average, at 3.8 months before and 4.8 months after birth, spaced approximately 21 to 25 days apart (Table 2).

**Table 2.** Frequency, Spacing, and Timing of Mental Health Assessments and Visits

Sample	Prenatal				Postpartum			
	No. of encounters		Spacing, mean (SD), d	Encounter date, mean (SD), No. of d after delivery	No. of encounters		Spacing, mean (SD), d	Encounter date, mean (SD), No. of d after delivery
	Mean (SD)	Median (IQR)			Mean (SD)	Median (IQR)		
Assessments								
PHQ-9	2.3 (2.7)	1 (1-2)	27.9 (30.0)	−144.9 (95.8)	3.4 (4.2)	1 (1-4)	26.9 (24.4)	164.8 (101.9)
EPDS	2.1 (1.5)	2 (1-3)	64.0 (47.8)	−93.7 (58.7)	1.7 (2.4)	1 (1-1)	34.1 (47.1)	92.9 (84.3)
GAD-7	4.4 (4.1)	3 (1-6)	21.1 (15.6)	−117.4 (95.0)	4.9 (5.0)	3 (1-7)	25.0 (22.0)	146.8 (95.7)
Visits								
Overall	6.4 (9.5)	2 (1-8)	27.4 (28.1)	−155.6 (100.6)	6.0 (12.9)	2 (1-6)	31.3 (29.8)	158.5 (104.9)
PHQ-9	8.4 (8.5)	6 (3-11)	23.2 (21.5)	−162.2 (96.0)	7.0 (7.9)	5 (2-9)	23.3 (20.2)	147.7 (104.5)
EPDS	5.7 (7.6)	2 (1-7)	30.4 (35.1)	−141.7 (89.2)	4.2 (7.1)	1 (1-3)	37.4 (39.0)	146.5 (107.7)
GAD-7	6.8 (7.7)	4 (2-9)	28.8 (31.8)	−156.0 (94.4)	5.4 (6.6)	2 (1-7)	26.2 (24.4)	137.9 (102.1)

Abbreviations: EPDS, Edinburgh Postnatal Depression scale; GAD-7, Generalized Anxiety Disorder–7 scale; PHQ-9, Patient Health Questionnaire–9 scale.

Overall, among women who were screened, 245 (51.9%) had clinically meaningful anxiety. Women reported high baseline anxiety (first GAD-7 administration score, mean [SD], 9.01 [5.54]) and peak anxiety (most severe GAD-7 score, mean [SD], 10.35 [5.65]).

We found that 23.2% (95% CI, 21.7%-24.8%) of women had clinically meaningful depression (EPDS, 24.0%; 95% CI, 22.3%-25.7%; PHQ-9, 21.3%; 95% CI, 18.4%-24.5%). Baseline depression severity was low to moderate (mean [SD] scores, PHQ-9, 4.93 [4.96]; EPDS, 5.53 [4.70]), with low-to-moderate peak severity (mean [SD] scores, PHQ-9, 5.59 [5.57]; EPDS, 6.52 [5.17]) across all women screened. Notably, prevalence estimates reflect each woman’s peak severity score across the study period, which may explain the relatively low mean scores. We also found that 8.8% (95% CI, 7.2%-10.8%) of women screened reported suicidal ideation, across both PHQ-9 (5.9%; 95% CI, 4.4%-8.0%) and EPDS (14.9%; 95% CI, 11.6%-19.0%) measures.

**Figure 2.**
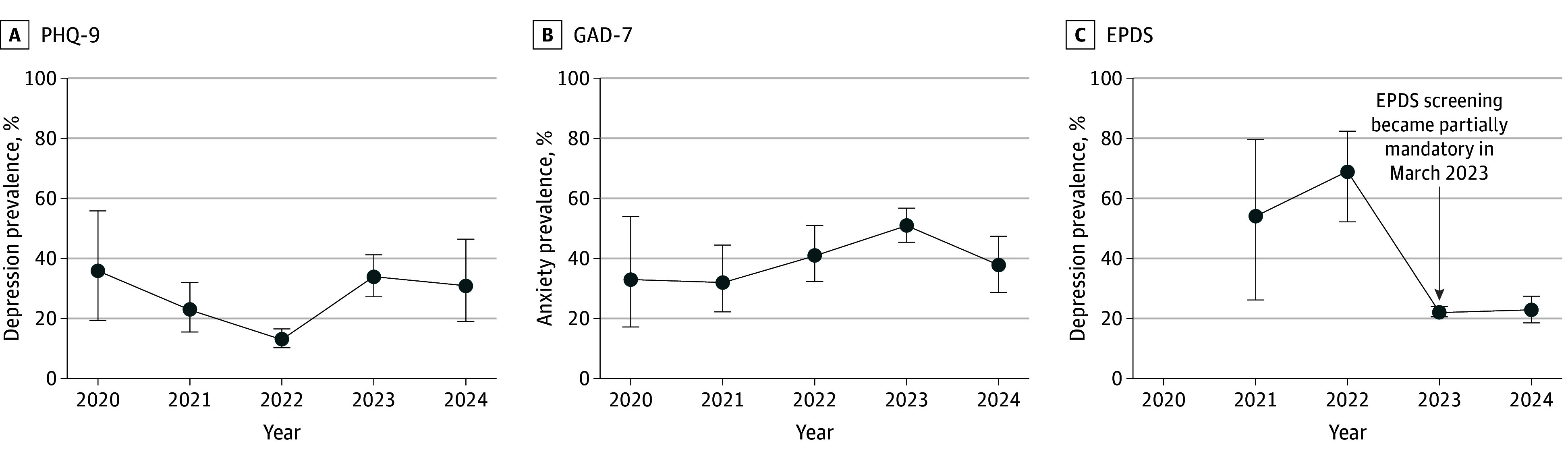
Prevalence of Clinically Meaningful Symptoms Among Women Screened During the Study Graphs show yearly prevalence of serious symptoms (score of ≥10) on each measure. Arrow in panel C marks policy change in obstetric and gynecological services requiring mandatory Edinburgh Postnatal Depression Scale (EPDS) screening in 3 clinics in the hospital. Error bars represent the 95% CIs around the point estimates for prevalence. GAD-7 indicates Generalized Anxiety Disorder–7; and PHQ-9, Patient Health Questionnaire–9.

Before March 2023, rates of clinically meaningful symptoms were 16.9% (95% CI, 14.0%-20.2%) for depression (PHQ-9), 29.9% (95% CI, 24.6%-35.8%) for perinatal depression (EPDS), and 45.1% (95% CI, 38.3%-52.0%) for anxiety (GAD-7), compared with 32.8% (95% CI, 26.0%-40.3%) for depression (PHQ-9), 23.0% (95% CI, 21.3%-24.8%) for perinatal depression (EPDS), and 52.1% (95% CI, 46.4%-57.7%) for anxiety (GAD-7) after March 2023 (Figure 2 and eFigure 2 in Supplement 1). Taken together, after eliminating selection bias with mandatory screening, PHQ rates doubled, EPDS rates reduced slightly, anxiety remained steady, with approximately 50% of women experiencing clinically meaningful symptoms.

### Mental Health Services

Among women who were screened, 523 (17.1%) had at least 1 mental health visit and 224 (7.3%) had 2 to 10 visits (Table 1). Women who received mental health services attended a mean (SD) of 6.4 (9.5) prenatal visits and 6.0 (12.9) postpartum visits, 23 to 37 days apart, occurring approximately 5 months before and after birth (Table 2).

### Trajectories of Severity Over Time

**Figure 3.**
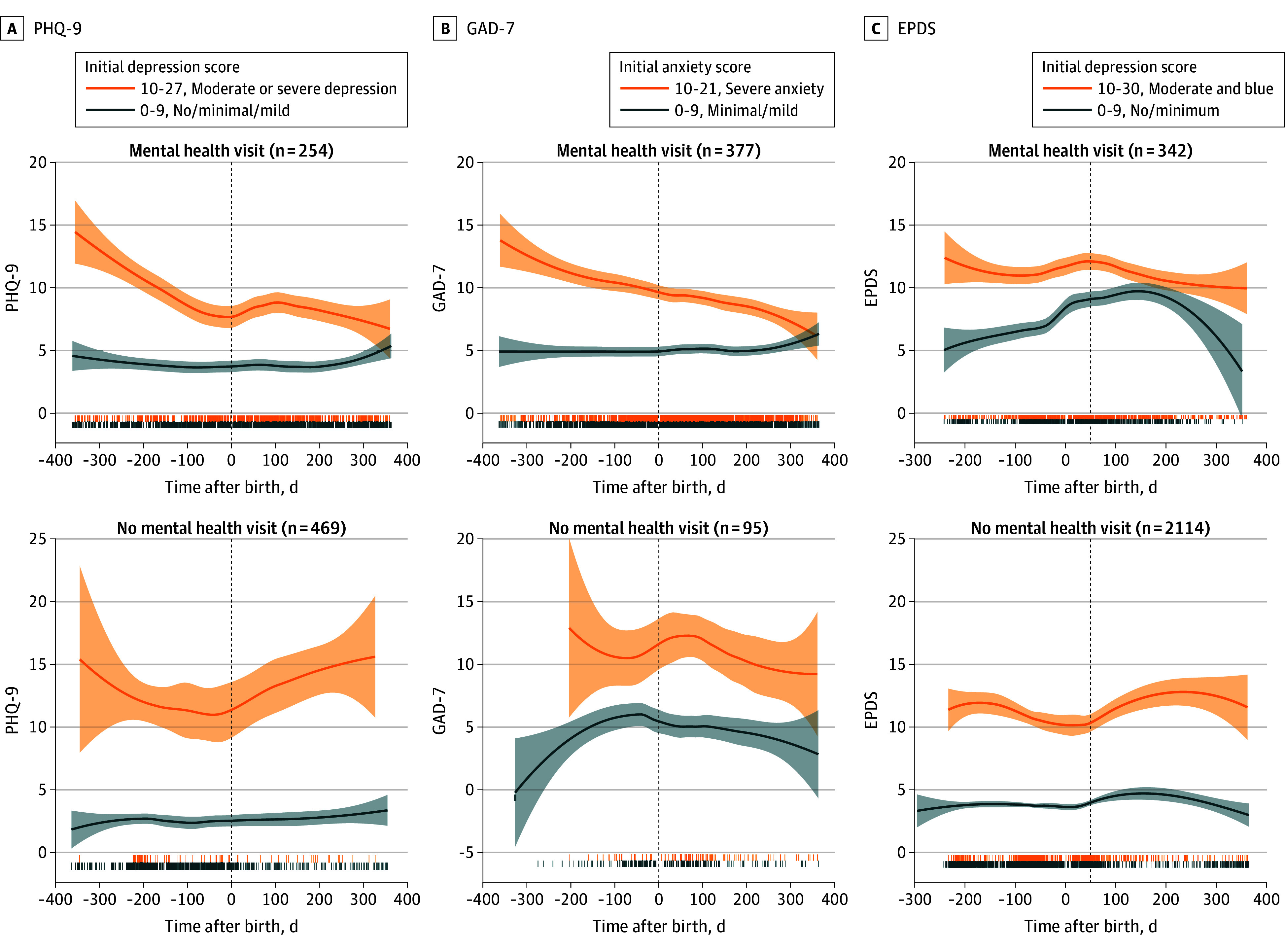
Trajectories of Change in Severity Over Time in Perinatal Depression and Anxiety for Women With and Without Mental Health Services Graphs show trajectories of change in severity over time, within 1 year before and after birth for women who received mental health services (ie, at least 1 mental health visit), and women who did not receive any services. Orange lines show trajectories for women with clinically meaningful symptoms; blue lines show women with minimal to no symptoms. Shaded areas around lines show 95% CIs. For the trajectory figures, a locally estimated scatterplot smoothing curve was fit to the raw data using a quadratic local polynomial and a smoothing span of 0.75. EPDS indicates Edinburgh Postnatal Depression Scale; GAD-7, Generalized Anxiety Disorder–7; and PHQ-9, Patient Health Questionnaire–9.

In mixed-effects models, we examined trajectories of change in depression and anxiety severity over time in women who were screened more than once (Figure 3). Women who received mental health services had faster reduction in depression severity over time (PHQ-9, *F*_1,1504_ = 9.6; *P* = .002) and higher overall depression severity (*F*_1,717_ = 32.1; *P* < .001) (eTable 3 in Supplement 1).

Perinatal depression severity (EPDS) followed a U-shaped curvilinear relationship (*F*_1,5162_ = 17.1; *P* < .001), with different trajectories for treated vs untreated women (*F*_1,5166_ = 33.8; *P* < .001) (eTable 4 in Supplement 1). Women who received mental health services showed increase in symptoms through late pregnancy, peaking at 2 months postpartum, followed by steady and continuous improvement. In contrast, untreated women showed early symptom decline during pregnancy to 4 months postpartum followed by worsening symptoms.

Anxiety severity assessed by GAD-7 followed a U-shaped curvilinear relationship (*F*_1,2019_ = 13.7; *P* < .001), with an initial increase followed by a decrease, with more gradual trajectory for women who received mental health services compared with those who did not (*F*_1,2017_ = 6.2; *P* = .01) (eTable 5 in Supplement 1). Anxiety severity peaked earlier among untreated women (0.3 months prenatal) than those who were treated (7.2 months prenatal).

## Discussion

The findings of this cohort study highlight the need for routine screening and treatment for perinatal depression and anxiety. Only 2.0% to 3.0% of women were screened using the PHQ-9 and GAD-7. After implementing mandatory EPDS screening in 3 clinics (covering 35% of births), rates increased 14-fold, from 1.0% to 14.2%. These rates were lower than expected because of early automation issues that resolved after 6 months. Thus, this increase reflects a rapid meaningful improvement, demonstrating that mandatory screening can quickly enhance detection of mental health risks in perinatal women.^15,37^ However, the results also show that implementation requires time, resources, staff education, and compliance monitoring.^38,39^ This pilot program was successful and, since study completion, has expanded to all clinics, integrated with a mental health referral system. Together, a streamlined screening and intervention system can improve maternal and infant outcomes.

Women were screened 1 to 3 times, most often once during the perinatal period. Assessments occurred 4 to 5 months before and after birth, with approximately 2 months between screens. Mandatory screening required EPDS administration at 3 key perinatal points, following American College of Obstetricians and Gynecologists guidelines.^5^ Our findings highlight ongoing challenges in routine monitoring for perinatal depression and anxiety, even in well-supported environments. They also underscore the need for routine screening around birth and during pediatric visits to improve risk detection and support early intervention.^37^

We found that 23.2% of women endorsed clinically meaningful perinatal depression, consistent with prior reports,^40^ and 8.8% reported suicidality. Suicidality during the perinatal period is associated with poor birth outcomes, disrupted maternal-infant bonding, negative parenting behaviors, and maternal mortality.^41^ However, these rates may still underestimate the true burden, as perinatal women often underreport suicidality because of concerns about stigma, involuntary hospitalization, or custody loss.^42^ These findings underscore the critical need for routine perinatal suicide risk assessment and timely interventions for perinatal depression and suicidality.^43,44^

Mandatory screening increases the likelihood that women receive mental health care, improving long-term outcomes.^15,37^ In our sample of screened women, 17.1% received services, typically monthly brief interventions (approximately 6 prenatal and 6 postpartum sessions), beginning approximately 4 months before and after delivery, indicating limited support near childbirth. These women showed faster symptom reduction and continuous improvement, compared with untreated women, suggesting sustained benefits. Data on treatment types and pharmacotherapy use were unavailable. However, our findings support the protective role of interventions against persistent postpartum depression.^45,46^ Scalable interventions, such as smartphone application–based approaches^47^ and behavioral activation models,^48^ can be especially efficacious. For example, we developed Engage & Connect, a brief psychotherapy targeting the reward system by increasing participation in pleasurable social activities.^49,50,51^

Most women were screened after mandatory EPDS implementation, which required completing the EPDS before or during visits. This universal approach likely increased sample diversity and generalizability. Furthermore, increased diversity in our sample may reflect an increase in practitioner diversity at pilot clinics over the study period, as prior work suggests that patients prefer practitioners from similar racial and ethnic backgrounds.^52^ Universal screening likely reduced practitioner biases, which has been shown to contribute to the minimization and underdiagnosis of mental health symptoms reported by underserved patients.^53^

### Limitations

Our study has several limitations. EHR data are inherently limited by inconsistent documentation and missing data, and our prevalence estimates are based on a subset of 3051 women who completed screens. Estimates before mandatory EPDS implementation may be inflated because of selective screening based on clinical judgment. However, most women were screened after implementation, enhancing generalizability. Thus, this study provides current data on perinatal severity trajectories in diverse clinical practice settings and can inform intervention development. Additional limitations include missing data from paper-based screens, lack of key risk factors (eg, sexual orientation, gender identity, substance use, or social stressors), and specific treatments. Some women likely received mental health services outside of the system that were not captured. Future work could address these gaps using artificial intelligence–driven EHR analyses.^31,32^ Despite limitations, our study offers important insights into perinatal mental health in a large diverse urban hospital system.

## Conclusions

In conclusion, we found that most women are not screened for mental health problems during the perinatal period. When women are screened, depression symptoms are common, with approximately 1 in 5 women reporting clinically meaningful symptoms and nearly 9% reporting suicidality. When women are treated, they experience rapid reductions in depression severity with sustained decline later in the postpartum period. Together, our results demonstrate the imperative to routinely screen, diagnose, and treat women in the perinatal period. Routine mandatory screening and treatment can enhance risk detection, reduce persistence of symptoms, and improve long-term outcomes for mothers and infants.

## Notes

Supplement 1. **eFigure 1.** Changes in prevalence of significant anxiety and depression before vs. after March 2023 **eTable 1.** Demographics, mental health visits, and delivery type for the sample at delivery (“baseline”) before vs. after March 2023 **eTable 2.** Demographics, mental health visits and delivery type for the sample at delivery (“baseline”) for included vs. excluded sample due to lack of mental health screenings **eFigure 2.** Changes in symptom severity over time pre-March 2023 vs post-March 2023 **eTable 3.** Mixed-effects model for depression severity trajectories over time (PHQ-9) **eTable 4.** Mixed-effects model for perinatal depression severity trajectories over time (EPDS) **eTable 5.** Mixed-effects model for perinatal anxiety severity trajectories over time (GAD-7)

Supplement 2.
Data Sharing Statement
