# Supplementary material for: Prevalence and Trajectories of Perinatal Anxiety and Depression in a Large Urban Medical Center
Source: JAMA Netw Open. 2025 Sep 22;8(9):e2533111. doi: 10.1001/jamanetworkopen.2025.33111 (PMC12455385; doi:10.1001/jamanetworkopen.2025.33111)
Supplement: Supplement 1. — eFigure 1. Changes in prevalence of significant anxiety and depression before vs. after March 2023 eTable 1. Demographics, mental health visits, and delivery type for the sample at delivery (“baseline”) before vs. after March 2023 eTable 2. Demographics, mental health visits and delivery type for the sample at delivery (“baseline”) for included vs. excluded sample due to lack of mental health screenings eFigure 2. Changes in symptom severity over time pre-March 2023 vs post-March 2023 eTable 3. Mixed-effects model for depression severity trajectories over time (PHQ-9) eTable 4. Mixed-effects model for perinatal depression severity trajectories over time (EPDS) eTable 5. Mixed-effects model for perinatal anxiety severity trajectories over time (GAD-7) [file jamanetwopen-e2533111-s001.pdf]

Supplemental Online Content

Solomonov N, Kerchner D, Dai Y, et al. Prevalence and trajectories of perinatal anxiety and depression in a large urban medical center. *JAMA Netw Open*. 2025;8(9):e2533111. doi:10.1001/jamanetworkopen.2025.33111

eFigure 1. Changes in prevalence of significant anxiety and depression before vs. after March 2023

eTable 1. Demographics, mental health visits, and delivery type for the sample at delivery (“baseline”) for included vs. excluded sample due to lack of mental health screenings

eTable 2. Demographics, mental health visits, and delivery type for the sample at delivery (“baseline”) before vs. after March 2023

eFigure 2. Changes in symptom severity over time before vs. after March 2023

eTable 3. Mixed-effects model for depression severity trajectories over time (PHQ-9)

eTable 4. Mixed-effects model for perinatal depression severity trajectories over time (EPDS)

eTable 5. Mixed-effects model for perinatal anxiety severity trajectories over time (GAD-7)

This supplemental material has been provided by the authors to give readers additional information about their work.

© 2025 Solomonov N et al. *JAMA Network Open*.

eFigure 1. Changes in prevalence of significant anxiety and depression before vs. after March 2023

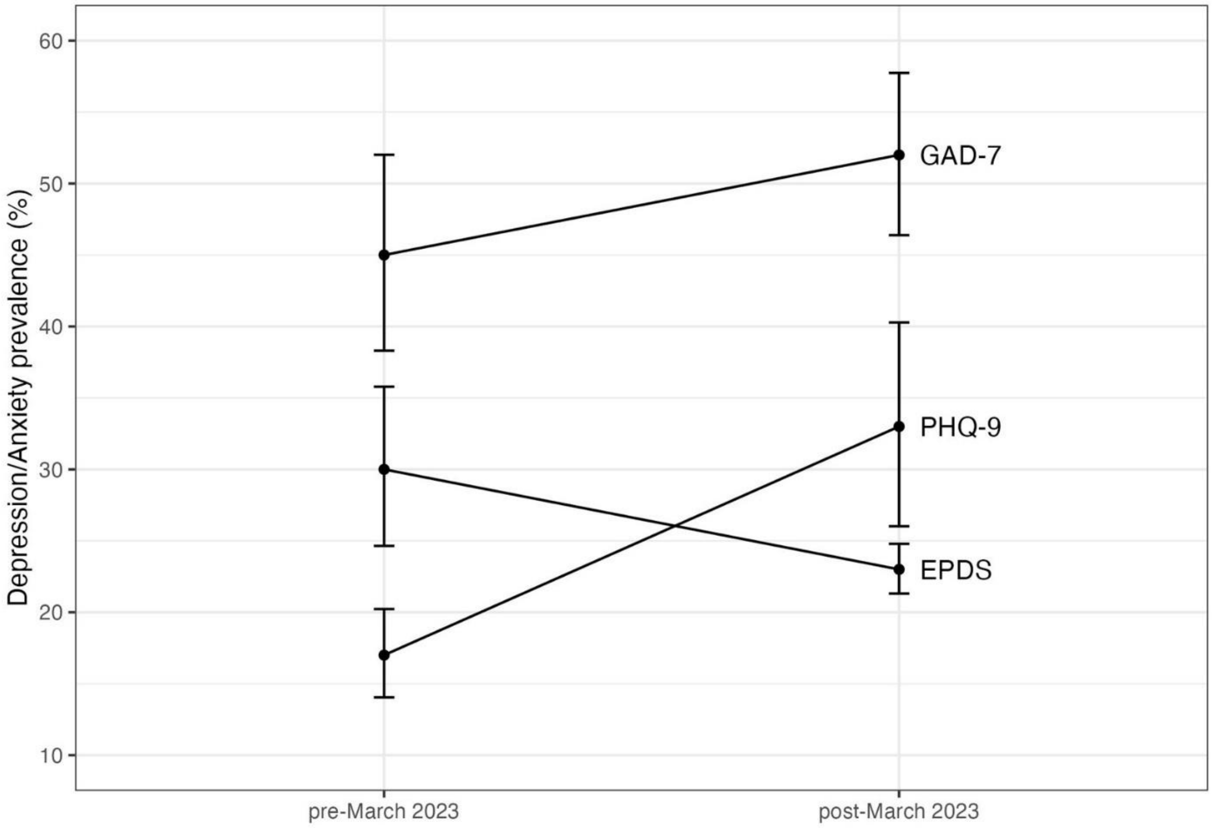

Note. Change in significant depression and anxiety (GAD-7, EPDS, PHQ score ≥10) prevalence (i.e. percent of women out of those screened), pre- and post-March after policy change in OBGYN services requiring mandated EPDS screening. Bars represent 95% confidence intervals; GAD-7 = Generalized Anxiety Disorder-7 Scale; EPDS = Edinburgh Postnatal Depression Scale; PHQ-9 = Patient Health Questionnaire-9 Scale.

© 2025 Solomonov N et al. *JAMA Network Open*.

eTable 1. Demographics, mental health visits and delivery type for the sample at delivery (“baseline”) for included vs. excluded sample due to lack of mental health screenings

**Variable**	**Excluded** N = 24,342[1]	**Included** N = 3,051[1]	**p-value**[2]
Race			<0.001
American Indian or Alaska Native	34 (0.2%)	12 (0.4%)	
Asian	4,496 (21%)	497 (17%)	
Black or African American	1,422 (6.6%)	321 (11%)	
Native Hawaiian/Other Pacific Islander	18 (<0.1%)	0 (0%)	
White	11,463 (53%)	1,506 (51%)	
Other[3]	1,924 (8.9%)	399 (13%)	
Declined	2,251 (10%)	222 (7.5%)	
Unknown	2,734	94	
Ethnicity			<0.001
Hispanic or Latina	2,008 (9.3%)	482 (16%)	
Not Hispanic or Latina	19,600 (91%)	2,475 (84%)	
Unknown	2,734	94	
Marital Status			<0.001
Married	18,620 (87%)	2,329 (79%)	
Single	1,790 (8.3%)	336 (11%)	
Domestic Partner/Significant Other	944 (4.4%)	268 (9.1%)	
Divorced/Separated	68 (0.3%)	9 (0.3%)	
Other	71 (0.3%)	4 (0.1%)	
Unknown	2,849	105	
Age[4]	34.8 (4.5)	34.3 (5.2)	<0.001
Unknown	2,734	94	
Number of mental health visits			<0.001
0	23,723 (97%)	2,528 (83%)	
1	322 (1.3%)	179 (5.9%)	
2-10	197 (0.8%)	224 (7.3%)	
11-30	59 (0.2%)	105 (3.4%)	
>30	41 (0.2%)	15 (0.5%)	
Delivery Type[5]			<0.001
C-Section	7,791 (32%)	1,145 (38%)	
SAB/TAB	17 (<0.1%)	4 (0.1%)	
Vacuum	42 (0.2%)	16 (0.5%)	
Vaginal, Instrument-Assisted	451 (1.9%)	48 (1.6%)	
Vaginal, Spontaneous	15,710 (65%)	1,797 (59%)	
VBAC	295 (1.2%)	40 (1.3%)	
Unknown	36	1	

© 2025 Solomonov N et al. *JAMA Network Open*.

Note. [1]n (%); Mean (SD); [2]Pearson's Chi-squared test; Wilcoxon rank sum test; Fisher's exact test; [3]Includes Ashkenazi Jewish, Native Hawaiian/Other Pacific Islander, and Other Combinations Not Described; [4]Age at first birth in the study period; [5]SAB = spontaneous abortion; TAB = therapeutic abortion; VBAC = vaginal birth after cesarean. For multiple birth deliveries (n=7) with differing delivery types, one delivery type was arbitrarily chosen for reporting.

© 2025 Solomonov N et al. *JAMA Network Open*.

eTable 2. Demographics, mental health visits, and delivery type for the sample at delivery ("baseline") before vs. after March 2023 for women who completed the EPDS

**Variable**	**Pre-March 2023** N = 274[1]	**Post-March 2023** N = 2,304[1]	**p-value**[2]
Race			0.009
American Indian or Alaska Native	2 (0.7%)	8 (0.4%)	
Asian	63 (23%)	397 (18%)	
Black or African American	19 (7.0%)	228 (10%)	
White	151 (56%)	1,181 (53%)	
Other[3]	25 (9.3%)	265 (12%)	
Declined	10 (3.7%)	165 (7.4%)	
Unknown	4	60	
Ethnicity			0.018
Hispanic or Latino	24 (8.9%)	317 (14%)	
Not Hispanic or Latino	246 (91%)	1,927 (86%)	
Unknown	4	60	
Marital Status			0.10
Married	237 (88%)	1,825 (82%)	
Single	18 (6.7%)	210 (9.4%)	
Domestic Partner/Significant Other	14 (5.2%)	192 (8.6%)	
Divorced/Separated	1 (0.4%)	5 (0.2%)	
Other	0 (0%)	3 (0.1%)	
Unknown	4	69	
Age[4]	35.5 (4.6)	34.6 (5.1)	0.008
Unknown	4	60	
Number of mental health visits			<0.001
0	204 (74%)	1,994 (87%)	
1	23 (8.4%)	132 (5.7%)	
2-10	24 (8.8%)	123 (5.3%)	
11-30	20 (7.3%)	45 (2.0%)	
>30	3 (1.1%)	10 (0.4%)	
Delivery Type[5]			0.20
C-Section	97 (35%)	899 (39%)	
SAB/TAB	0 (0%)	3 (0.1%)	
Vacuum	2 (0.7%)	15 (0.7%)	
Vaginal, Instrument-Assisted	9 (3.3%)	31 (1.3%)	
Vaginal, Spontaneous	163 (59%)	1,327 (58%)	
VBAC	3 (1.1%)	29 (1.3%)	

© 2025 Solomonov N et al. *JAMA Network Open*.

Note. [1]n (%); Mean (SD); [2]Pearson's Chi-squared test; Wilcoxon rank sum test; Fisher's exact test; [3]Includes Ashkenazi Jewish, Native Hawaiian/Other Pacific Islander, and Other Combinations Not Described; [4]Age at first birth in the study period; [5]SAB = spontaneous abortion; TAB = therapeutic abortion; VBAC = vaginal birth after cesarean. For multiple birth deliveries (n=7) with differing delivery types, one delivery type was arbitrarily chosen for reporting.

© 2025 Solomonov N et al. *JAMA Network Open*.

eFigure 2. Changes in symptoms over time pre-March 2023 vs post-March 2023

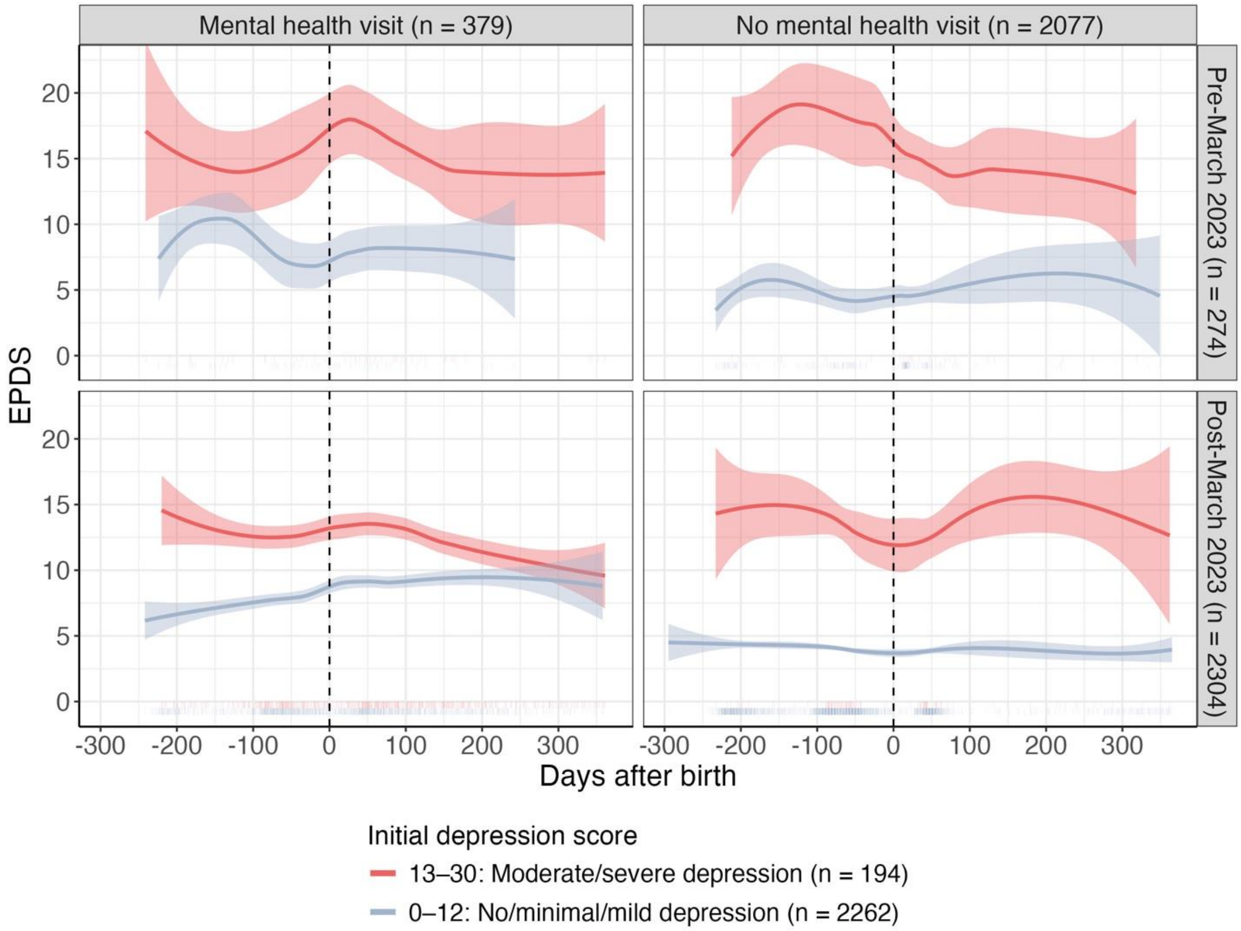

Note. EPDS = Edinburgh Postnatal Depression Scale. Trajectories of change in severity over time, within one year before and after birth for women who received mental health services (i.e. at least one mental health visit), and women who did not receive any services. Red lines show trajectories for women with clinically significant symptoms; blue lines show women with minimal to no symptoms. All lines show 95% confidence interval. For the trajectory figures a locally estimated scatterplot smoothing (LOESS) curve was fit to the raw data using a quadratic local polynomial and a smoothing span of 0.75

© 2025 Solomonov N et al. *JAMA Network Open*.

eTable 3. Mixed-effects model for depression severity trajectories over time (PHQ-9)

Variable	N	Beta	95% CI	p-value
Months from birth (linear term)	2,088	0.039	-0.030, 0.109	0.26
Mental health visit	2,088			
FALSE		—	—	
TRUE		2.20	1.44, 2.97	<0.001
Race	2,088			
White		—	—	
Asian		0.604	-0.381, 1.59	0.23
Black or African American		1.04	-0.053, 2.12	0.062
Declined		0.859	-0.373, 2.09	0.17
Other		-0.226	-1.42, 0.965	0.71
Marital Status	2,088			
Married/Partnered		—	—	
Other		-0.207	-1.13, 0.717	0.66
Ethnicity	2,088			
Not Hispanic or Latino		—	—	
Hispanic or Latino		0.709	-0.323, 1.74	0.18
Age	2,088	0.054	-0.012, 0.120	0.11
Months from birth (linear term) * Mental health visit	2,088			
Months from birth (linear term) * TRUE		-0.135	-0.220, -0.049	0.002

Note. PHQ-9 = Patient Health Questionnaire – 9; CI = Confidence Interval

© 2025 Solomonov N et al. *JAMA Network Open*.

eTable 4. Mixed-effects model for perinatal depression severity trajectories over time (EPDS)

Variable	N	Beta	95% CI	p-value
Months from birth (linear term)	5,844	-0.034	-0.071, 0.002	0.064
Months from birth (quadratic term)	5,844	0.004	-0.001, 0.010	0.14
Mental health visit	5,844			
FALSE		—	—	
TRUE		4.94	4.46, 5.42	<0.001
Race	5,844			
White		—	—	
Asian		1.11	0.661, 1.56	<0.001
Black or African American		0.552	-0.035, 1.14	0.065
Declined		0.344	-0.312, 1.00	0.3
Other		0.915	0.235, 1.60	0.008
Marital Status	5,844			
Married/Partnered		—	—	
Other		0.233	-0.349, 0.816	0.4
Ethnicity	5,844			
Not Hispanic or Latino		—	—	
Hispanic or Latino		-0.187	-0.813, 0.440	0.6
Age	5,844	0.013	-0.020, 0.047	0.4
Months from birth (linear term) * Mental health visit	5,844			
Months from birth (linear term) * TRUE		0.035	-0.032, 0.102	0.3
Months from birth (quadratic term) * Mental health visit	5,844			
Months from birth (quadratic term) * TRUE		-0.029	-0.039, -0.019	<0.001

Note. EPDS = Edinburgh Postnatal Depression Scale; CI = Confidence Interval

© 2025 Solomonov N et al. *JAMA Network Open*.

eTable 5. Mixed-effects model for perinatal anxiety severity trajectories over time (GAD-7)

Variable	N	Beta	95% CI	p-value
Months from birth (linear term)	2,671	-0.028	-0.209, 0.154	0.77
Months from birth (quadratic term)	2,671	-0.041	-0.066, -0.016	0.001
Mental health visit	2,671			
FALSE		—	—	
TRUE		-0.178	-1.45, 1.10	0.78
Race	2,671			
White		—	—	
Asian		0.974	-0.141, 2.09	0.087
Black or African American		1.97	0.338, 3.61	0.018
Declined		0.739	-0.900, 2.38	0.38
Other		0.268	-1.55, 2.09	0.77
Marital Status	2,671			
Married/Partnered		—	—	
Other		-0.616	-2.33, 1.10	0.48
Ethnicity	2,671			
Not Hispanic or Latino		—	—	
Hispanic or Latino		0.661	-0.955, 2.28	0.42
Age	2,671	-0.031	-0.125, 0.062	0.51
Months from birth (linear term) * Mental health visit	2,671			
Months from birth (linear term) * TRUE		-0.089	-0.277, 0.099	0.35
Months from birth (quadratic term) * Mental health visit	2,671			
Months from birth (quadratic term) * TRUE		0.033	0.007, 0.059	0.013

Abbreviation: CI = Confidence Interval

Note. GAD-7 = Generalized Anxiety Disorder Scale-7

© 2025 Solomonov N et al. *JAMA Network Open*.
