# Supplementary material for: Prevalence and Trajectories of Perinatal Anxiety and Depression in a Large Urban Medical Center
Source: JAMA Netw Open. 2025 Sep 22;8(9):e2533111. doi: 10.1001/jamanetworkopen.2025.33111 (PMC12455385; doi:10.1001/jamanetworkopen.2025.33111)
Supplement: Supplement 2. — Data Sharing Statement [file jamanetwopen-e2533111-s002.pdf]

Data Sharing Statement

Solomonov. Prevalence and Trajectories of Perinatal Anxiety and Depression in a Large Urban Medical Center. *JAMA Netw Open*. Published September 22, 2025.
doi:10.1001/jamanetworkopen.2025.33111

Data
Data available: No

Additional Information
Explanation for why data not available: Data will be made available upon request from the authors and in compliance with the IRB's policy.
